# Promoter methylation of tumor suppressor genes in pre-neoplastic lesions; potential marker of disease recurrence

**DOI:** 10.1186/s13046-014-0065-x

**Published:** 2014-08-05

**Authors:** Claudia Rengucci, Giulia De Maio, Andrea Casadei Gardini, Mattia Zucca, Emanuela Scarpi, Chiara Zingaretti, Giovanni Foschi, Maria Maddalena Tumedei, Chiara Molinari, Luca Saragoni, Maurizio Puccetti, Dino Amadori, Wainer Zoli, Daniele Calistri

**Affiliations:** 1 Biosciences Laboratory, Istituto Scientifico Romagnolo per lo Studio e la Cura dei Tumori (IRST) IRCCS, Meldola, Italy; 2 Department of Medical Oncology, Istituto Scientifico Romagnolo per lo Studio e la Cura dei Tumori (IRST) IRCCS, Meldola, Italy; 3 Unit of Biostatistics and Clinical Trials, Istituto Scientifico Romagnolo per lo Studio e la Cura dei Tumori (IRST) IRCCS, Meldola, Italy; 4 National Institute of Molecular Genetics, Milan, Italy; 5 Pathology Unit, Morgagni-Pierantoni Hospital, Forlì, Italy; 6 Pathology Unit, Santa Maria delle Croci Hospital, Ravenna, Italy

**Keywords:** Colorectal adenoma, Gene promoter methylation profile, Risk of recurrence, Prognostic marker, Pre-neoplastic lesion classification

## Abstract

**Background:**

Epigenetic alterations of specific genes have been reported to be related to colorectal cancer (CRC) transformation and would also appear to be involved in the early stages of colorectal carcinogenesis. Little data are available on the role of these alterations in determining a different risk of colorectal lesion recurrence. The aim of the present study was to verify whether epigenetic alterations present in pre-neoplastic colorectal lesions detected by colonoscopy can predict disease recurrence.

**Methods:**

A retrospective series of 78 adenomas were collected and classified as low (35) or high-risk (43) for recurrence according to National Comprehensive Cancer Network guidelines. Methylation alterations were analyzed by the methylation-specific multiplex ligation probe assay (MS-MLPA) which is capable of quantifying methylation levels simultaneously in 24 different gene promoters. MS-MLPA results were confirmed by pyrosequencing and immunohistochemistry.

**Results:**

Higher levels of methylation were associated with disease recurrence. In particular, *MLH1*, *ATM* and *FHIT* gene promoters were found to be significantly hypermethylated in recurring adenomas. Unconditional logistic regression analysis used to evaluate the relative risk (RR) of recurrence showed that *FHIT* and *MLH1* were independent variables with an RR of 35.30 (95% CI 4.15-300.06, *P* = 0.001) and 17.68 (95% CI 1.91-163.54, *P* = 0.011), respectively.

**Conclusions:**

Histopathological classification does not permit an accurate evaluation of the risk of recurrence of colorectal lesions. Conversely, results from our methylation analysis suggest that a classification based on molecular parameters could help to define the mechanisms involved in carcinogenesis and prove an effective method for identifying patients at high risk of recurrence.

## Background

Colorectal cancer (CRC), a disease arising from complex and heterogeneous etiological factors and pathogenetic mechanisms, develops in a multi-step manner from normal epithelium, through a pre-malignant lesion (adenoma), into a malignant lesion (carcinoma) [1]. Histopathological evaluation of early stage CRC in many cases reveals areas of adenomatous mucosa, but the presence of tissue with histological features ranging from pure tubular to pure villous adenomas accompanied by dysplasia is also frequently detected in invasive colorectal cancer [1],[2]. Although individuals with syndromes that strongly predispose to adenomas, *e.g.* familial adenomatous polyposis (FAP), invariably develop CRC by the third to fifth decade of life if these lesions are not removed [3], most adenomas (not FAP) have a low risk of progressing into cancer (about 5%) if not resected. An adenomatous polyp is a much more frequent finding than CRC and polypectomy has a distinctly protective effect on the subsequent development of CRC. It has been estimated that in the first 10 years after polypectomy, the risk of CRC is reduced to a level similar to that of individuals whose colonoscopy does not reveal the presence of polyps [4],[5].

Different molecular mechanisms seem to be related to CRC development. The vast majority of tumors (about 50-80%), present chromosomal instability (CIN) [3],[6],[7], while a smaller fraction (10-15%) is characterized by microsatellite instability (MSI) [3],[6],[7]. In recent years, epigenetic alterations have gained recognition as a key mechanism in carcinogenesis. In particular, hypermethylation of CpG islands present in gene promoter sequences leads to the inactivation of tumor suppressor genes, working in a different way with respect to genetic mutations [8],[9].

This aberrant methylation status occurs at the same time as genetic alterations which drive the initiation and progression of colorectal cancer, suggesting that methylation plays an important role in many stages of tumor transformation [10]-[14]. The existence of a methylator phenotype could be related to distinctive biological and/or clinical characteristics [15].

CRCs that show hypermethylation changes in numerous different CpG-rich DNA regions are defined as showing the CpG island methylator phenotype (CIMP) [16]. CIMP-positive cancers have distinct clinical pathological characteristics such as proximal colon location, mucinous and poorly differentiated histology, female preponderance and older age [17]. This phenotype also seems to be associated with MSI and *BRAF* mutations [18],[19]. Conversely, hypomethylation of specific sequences may decrease the fidelity of chromosomal segregation [20], suggesting that it may be involved in the chromosomal instability phenotype [21]. DNA methylation changes probably lead adenomatous precursor lesions to progress into malignant tumors. In fact, sessile serrated adenomas, considered important precursors of cancer, are often CIMP-positive.

Taking the above considerations into account, a better understanding of the epigenetic mechanisms associated with adenoma-carcinoma transition could represent an important tool for CRC prevention. In accordance with international guidelines, pre-neoplastic lesions of the colon and rectum are classified according to pathological parameters (size, histology, number of polyps and dysplasia) as having high or low risk of recurrence. In high risk patients a new colonoscopy is performed after 3 years, while in low risk subjects the time interval is extended to 5 years. However, this type of subdivision is unable to predict the real risk of developing a new lesion. In fact, it has been seen that patients who are classified as high risk may not experience any further problems, while those who are classed as low risk may relapse after a short time.

Little data is available on the relationship between risk of recurrence of pre-neoplastic lesions and molecular alterations of colorectal lesions, whereas a great deal is known about the mechanisms of CRC transformation. Although a number of gene promoter methylation profiles have been shown to characterize specific stages of tumor progression, no data are available on epigenetic alterations or risk of disease evolution/recurrence. The identification of these specific epigenetic profiles could help us to better understand the mechanisms of adenoma recurrence and, possibly, adenoma-carcinoma transition, resulting in a more accurate classification of the risk of recurrence of pre-neoplastic and permitting a personalized program of cancer prevention.

The aim of this study was to evaluate whether altered methylation profiles in pre-neoplastic lesions sampled by colonoscopy is capable of identifying patients at high risk of recurrence with greater accuracy than conventional clinical pathological parameters.

## Methods

### Case series

We evaluated formalin fixed paraffin-embedded (FFPE) tissue samples of pre-neoplastic colorectal lesions endoscopically identified and surgically removed from a series of 78 patients who underwent follow up for at least 5 years. Lesions were classified as adenomas at low risk (3 tubular polyps with a diameter < 1 cm) or high risk (high-risk dysplasia, > 3 adenomatous villous or tubulovillous polyps, at least one of which with a diameter of ≥ 1 cm, or an *in situ* carcinoma) of recurrence according to National Comprehensive Cancer Network guidelines. All tissue samples were obtained from the Pathology Unit of Morgagni-Pierantoni Hospital (Forlì, Italy). Informed consent for the use of biological samples was obtained from all individuals who agreed to take part in the study for research purposes. The study protocol was reviewed and approved by the IRST Ethics Committee.

### DNA extraction

DNA was extracted using a digestion buffer (50 mM KCl, 10 mM Tris–HCl pH8, 2.5 mM MgCl2, 0.45% v/v TWEEN-20 and proteinase K 25 mg/ml). Approximately three 5-μm slices of paraffin-embedded tissue was added to 150 ml of home-made buffer and 10 ml of proteinase K (25 mg/ml). After overnight incubation at 58°C with gentle shaking, the sample was heated to 98°C for 10 min, cooled to room temperature and then centrifuged at 6000 rpm for 10 min. The supernatant containing DNA was transferred to a new vial and centrifuged again as per the previous step until all traces of paraffin were removed. The quality and quantity of DNA were assessed using NanoDrop ND-1000 (Thermo Fisher Scientific, Waltham, USA) and the DNA was stored at −20°C until molecular analysis was performed.

### Quantitative DNA methylation analyses

#### Methylation-specific multiplex ligation probe analysis

Methylation-specific (MS) multiplex ligation probe analysis (SALSA MLPA ME001 Tumour Suppressor-1 kit, MLPA®; MRC-Holland, Amsterdam, The Netherlands), a high-throughput, semi-quantitative, methylation-specific enzyme-based polymerase chain reaction (PCR) assay, was performed according to the manufacturer’s instructions. Each probe corresponded to a single gene, apart from *MLH1* and *RASSF1*, both of which contained two probes. Two ligation probe reactions were needed to calculate the percentage of methylation, one of which contained the methylation-sensitive enzyme HhaI. Briefly, 200 ng of each sample was diluted to 5 μl with TE buffer and heated at 98°C for 10 min followed by incubation at 25°C for 5 min in a thermocycler. Following the addition of ligation probes, samples were first incubated at 98°C for 1 min and then at 60°C for 16–18 h to permit hybridization. Samples were split equally into two vials, each containing the same amount of DNA (volume 10 ul). Ligase-65 mix (Ligase-65 buffer, Ligase-65 enzyme and water) was added to the first vial, and Ligase-Digestion mix (Ligase-65 buffer, Ligase- 65 enzyme, HhaI enzyme [Promega, Southampton, UK] and water) to the second.

Both samples were incubated at 49°C for 30 min, after which the ligase enzyme was inactivated by heating at 98°C for 5 min. PCR buffer, deoxynucleoside 5-triphosphates (dNTPs) and Taq polymerase were added to the samples during preheating at 72°C. The PCR reaction was performed in a thermocycler preheated to 72°C, under the following conditions: 35 cycles at 95°C for 30 s, 60°C for 30 s and 72°C for 60 s. The final incubation was at 72°C for 20 min. Amplification products were analyzed on an ABI-3130 DNA Analyzer (Applied Biosystems, Warrington, UK). Negative water controls were included to ensure no contamination. Internal validation was performed using unmethylated and methylated genomic DNA (Millipore, Watford, UK).

Intrasample normalization was performed to address peak variations due to fluctuations in the assay run, such as amount of DNA, ploidy variations and PCR conditions, The relative peak height of each probe was determined by dividing the absolute peak height by the mean height of all 15 control probes. A methylation percentage for each probe was obtained using the following calculation, as described previously [22]:

(1) $$\mathrm{Methylation}\left(\%\right)=\frac{{\left(\mathrm{peak}\;\mathrm{height}\;\mathrm{of}\;a\;\mathrm{given}\;\mathrm{probe}/\mathrm{mean}\;\mathrm{height}\;\mathrm{of}\;\mathrm{control}\;\mathrm{probes}\right)}_{\mathrm{with}\;\mathrm{Hha}1}}{{\left(\mathrm{peak}\;\mathrm{height}\;\mathrm{of}\;a\;\mathrm{given}\;\mathrm{probe}/\mathrm{mean}\;\mathrm{height}\;\mathrm{of}\;\mathrm{control}\;\mathrm{probes}\right)}_{\mathrm{without}\;\mathrm{Hha}1}}\times 100$$

### Validation of MS-MLPA results

Validation of MS-MLPA results was only performed for the three most significant genes: *ATM, FHIT* and *MLH1. ATM* and *MLH1* were confirmed by pyrosequencing CpG analysis, while *FHIT* was validated by immunohistochemistry (IHC) staining.

Twenty microliters of extracted DNA were converted using Epitect Bisulphite kit (Qiagen, Hilden, Germany) in accordance with the “Sodium Bisulphite Conversion of Unmethylated Cytosines in DNA” protocol. Converted DNA was eluted in 20 μl of elution buffer. Five microliters of bisulphite-treated DNA were used to amplify the specific promoter regions of *ATM* and *MLH1* genes with primer sets designed to amplify the same CpG sites as those of the MS-MLPA approach. Primer sets for amplification and sequencing were designed by Diatech Pharmacogenetics (Jesi, Italy) (Table 1).

**Table 1:** **Validation of MS-MLPA results for** ***ATM, MLH1*** **and** ***FHIT***

**Gene**	**Method**	**Primer sequence/polyclonal antibody**	**No. samples examined**	**Overall concordance (%)**
*ATM*	Pyrosequencing CpG analysis	Fw: 5′-AGAAGTGGGAGTTGGGTAGTT-3′	77/78	**73**%
		Rv: 5′-biotinCTCCCCCCCCCTACCACTACACTC-3′		
		Seq: 5′-AGGAGGAGAGAGGAGT-3′		
*MLH1*	Pyrosequencing CpG analysis	Fw: 5′-biotinGGGAGGTAAGTTTAAGTGGAATAT-3′	72/78	**79**%
		Rv: 5′-CCAATCCCCACCCTAAAACCCTC-3′		
		Seq: 5′-CTAAACTCCCAAATAATAACCT-3′		
*FHIT*	Immunohistochemistry	Rabbit polyclonal anti-FHIT; clone PA1-37690; Thermo Scientific Pierce; working dilution: 1/200	57/78	**84**%

*Abbreviations*: *Fw* Forward Primer, *Rv* Reverse primer, *Seq* sequence analyzed.

Each PCR reaction was performed in a final volume of 50 μl containing 2 μl of each primer (5 μM), 1 μl of Takara dNTP mixture (10 mM of each dNTP) (Takara Bio Inc., Otsu, Japan), 1 μl of Takara 50 mM Mg^++^ solution (Takara Bio Inc.), 2.5 μl of EvaGreen™ Dye (20X), 10 μl of Takara 5X R-PCR Buffer (Mg^++^ free) (Takara Bio Inc.), 0.5 μl of Takara Ex Taq™ HS (5 U/μl) (Takara Bio Inc.), 26 μl of water and 5 μl of bisulphite-treated DNA. Amplification was done by quantitative Real Time PCR on Rotor Gene™ 6000 (Corbett Life Science, Cambridge, UK) equipped with Rotor Gene 6000 Series Software 1.7 Build 87. The cycling programme for *ATM* and *MLH1* consisted of one hold cycle at 95°C for 5 min, the second hold cycle at 72°C for 5 min, one pre-melting cycle at 65°C for 90 s and then one melting cycle from 65°C to 95°C with an increase of 1°C every 5 s, with fluorescence acquisition. Between the first two holding cycles there were 45 cycles. For *ATM* gene, these cycles consisted of: denaturation at 95°C for 30 s, annealing 56°C for 30 s and elongation 72°C for 20 s. For *MLH1*, the 45 cycles comprised denaturation at 95°C for 30 s, annealing at 56°C for 60 s and an elongation cycle at 72°C for 30 s.

Promoter CpG sites were analyzed by PyroQ-CpG™ 1.0.9 software (Biotage, Uppsala, Sweden) on Pyromark Q96 ID (Qiagen). 40 μl of PCR products were added to 37 μl of binding buffer and 3 μl of Sepharose beads and mixed at 1400 rpm for 10 min at room temperature. The Sepharose beads with single-stranded templates attached were released into a plate containing an annealing mixture composed of 38.4 μl of annealing buffer and 1.6 μl of the corresponding sequencing primers. All the experimental procedures were carried out according to the manufacturer’s instructions. We added water as negative control and universal methylated and unmethylated samples as positive control.

Four-μm-thick FFPE adenoma sections were used for immunodetection. Sections were deparaffinized in xylene and dehydrated in a graded alcohol series, according to the local protocol. Antigen retrieval was achieved by microwaving in 10 mM of sodium citrate buffer at pH 6 for 30 min. Sections were incubated with rabbit polyclonal anti-FHIT (clone PA1-37690; Thermo Fisher Scientific, Waltham, USA) at a 1/200 working dilution. From this point onwards, all the steps were performed automatically by Autostainer Plus Staining System (Dako Cytomatic, Glostrop, Denmark). LSAB protein block (Dako; Carpinteria, USA) was performed for 15 min. The staining of the primary antibody was performed for 130 min. Sections were immunostained with anti-rabbit biotinylated secondary antibody LSAB (Dako) for 10 min. Visualization was performed using DAB chromogen (Dako). Sections were counterstained with hematoxylin, dehydrated in the same graded alcoholic scale and mounted. On the basis of antibody datasheet instructions, negative and positive control sections were incubated with the secondary antibody in the presence or not of the primary antibody, respectively.

### Statistical analysis

In order to evaluate the correlation between methylation status and prognosis for adenoma/disease recurrence, patients were subdivided into relapsed (R) or not relapsed (NR) at 60 months of follow-up. The relationship between clinical pathological characteristics and patient status was analyzed using the chi-square test.

Methylation was evaluated as both a continuous variable and binary variable. In particular, a cut off of 20% of methylated DNA was used to classify a promoter as hypermethylated. Hypermethylation frequencies in NR and R samples were compared using Fisher’s exact test. The student’s *T* test was used to compare the mean methylation levels of NR and R samples. Methylation status of multiple genes was evaluated to determine the presence of hypermethylation. Its accuracy (the proportion of R and NR patients correctly identified by the hypermethylated profile) in detecting recurrent lesions using the defined hypermethylation cut off was expressed in terms of sensitivity (proportion of R patients correctly identified by the hypermethylated profile) and specificity (proportion of NR patients correctly identified by the hypermethylated profile) in relation to the total series. For both indicators, 95% confidence intervals (95% CI) were calculated. Logistic regression was used to analyze the Relative Risks (RR) and their 95% CI for patient status and methylation status as dichotomous variables. All analyses were performed using SAS Statistical software (version 9.3, SAS Institute, Cary, North Carolina, USA) or Graphpad Prism software version 5.0d. Statistical significance for all tests was taken as *P* < 0.05. The validation of the MS-MLPA results was done considering the results obtained by pyrosequencing CpG analysis and IHC considered as dichotomous variables.

## Results

### MS-MLPA analysis

Tissue specimens of adenomas from 78 patients were collected for methylation status analysis (Table 2). Patients were divided into relapsed (R) or not relapsed (NR) on the basis of disease recurrence at 5 years of follow up. In particular, 47 patients (27 with high risk and 20 with low risk adenomas) did not show disease recurrence (NR), while 31 patients (16 with high risk and 15 low risk adenomas) developed new colorectal lesions (R) during this period. No differences in terms of recurrence were noted on the basis of pathological classification (high or low risk adenoma) and no correlation was found between the grade of dysplasia and development of new lesions during follow up. Conversely, the site of the first lesion was significantly related to risk of disease relapse (*P* = 0.015).

**Table 2:** Clinical pathological characteristics of the case series

	**Total n (%)**	**Disease recurrence n (%)**	**No. of disease recurrence n (%)**	***P***
**Gender**				
Male	56 (71.8)	24 (77.4)	32 (68.1)	
Female	22 (28.2)	7 (22.6)	15 (31.9)	0.523
**Median age, years (range)**				
Male	61 (42–85)	64 (48–85)	61 (42–79)	0.263
Female	66 (40–81)	63 (51–72)	66 (40–81)	0.972
**Risk of recurrence**				
High risk	43 (55.1)	16 (51.6)	27 (57.4)	
Low risk	35 (44.9)	15 (48.4)	20 (42.6)	0.784
**Dysplasia**				
Low (low and medium) grade	61 (78.2)	26 (83.9)	35 (74.5)	
High grade	17 (21.8)	5 (16.1)	12 (25.5)	0.481
**Lesion dimension**				
0–0.9 cm	9 (11.5)	3 (9.7)	6 (12.8)	
≥ 1 cm	29 (37.2)	11 (35.5)	18 (38.3)	
Not specified	40 (51.3)	17 (54.8)	23 (48.9)	1.000
**Lesion localization**				
Ascending colon	19 (24.4)	10 (32.3)	9 (19.1)	
Descending colon	37 (47.4)	9 (29.0)	28 (59.6)	
Mixed	22 (28.2)	12 (38.7)	10 (21.3)	0.015
**Adenoma morphology**				
Tubular	46 (59.0)	19 (61.3)	27 (57.4)	
Villous	3 (3.8)	0	3 (6.4)	
Tubulovillous (mixed)	29 (37.2)	12 (38.7)	17 (36.2)	0.441

**Figure 1:**
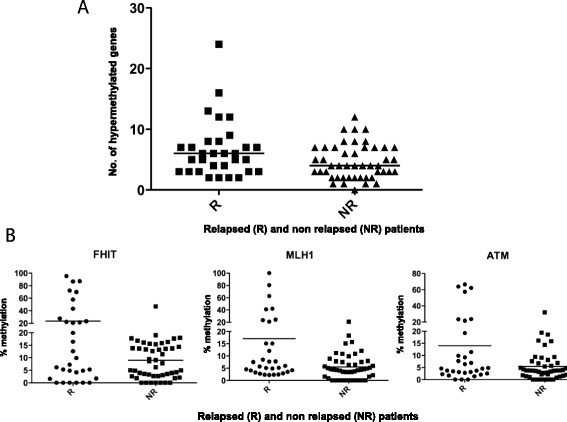
**Gene methylation level distribution. A)** Hypermethylated genes in the case series subdivided according to the presence or not of disease recurrence. **B)** Comparison of methylation levels of the three most significant genes in R and NR samples.

MS-MLPA analysis was performed for all samples, obtaining a quantification of methylation status for the entire case series. Two probes (GSTP1 and MLH1 CpG 02) were discarded from the analysis because they were negative for methylation (0% methylation level) in 92% and 83% of cases, respectively. We first evaluated the number of hypermethylated promoters in R and NR patients using a methylation level of 20% to define a gene promoter as hypermethylated. Primary lesions that relapsed showed a higher number of hypermethylated markers (median 6, range 2–24) than non recurring lesions (median 4, range 0–12) (Figure 1A).

The promoters of three genes (*FHIT, MLH1* and *ATM*) were found to be hypermethylated in a significantly higher fraction of adenomas that recurred compared to non recurring lesions (Figure 1B). Furthermore, *TP73* and *BRCA1* promoter hypermethylation were related to recurrence, albeit with low statistical significance or borderline significance, respectively (Table 3).

**Table 3:** Frequency of promoter hypermethylation in patients with recurrent or non recurrent disease

**Gene ID**	**% R**	**% NR**	**Overall series**	***P***
	**(Total = 31)**	**(Total = 47)**	**(Total = 78)**	
***FHIT***	38.71 (12/31)	2.13 (1/47)	16.67 (13/78)	3.1E-05
***MLH1***	25.81 (8/31)	2.13 (1/47)	11.54 (9/78)	0.002
***ATM***	22.58 (7/31)	2.13 (1/47)	10.26 (8/78)	0.006
***TP73***	35.48 (11/31)	12.77 (6/47)	21.79 (17/78)	0.025
***BRCA1***	9.68 (3/31)	0.00 (0/47)	3.85 (3/78)	0.059
***CHFR***	29.03 (9/31)	10.64 (5/47)	17.95 (14/78)	0.068
***IGSF4***	12.90 (4/31)	2.13 (1/47)	6.41 (5/78)	0.078
***ESR1***	70.97 (22/31)	85.11 (40/47)	79.49 (62/78)	0.158
***DAPK1***	22.58 (7/31)	10.64 (5/47)	15.38 (12/78)	0.203
***CDKN2B***	45.16 (14/31)	29.79 (14/47)	35.90 (28/78)	0.228
***RASSF1*** *CpG1*	41.94 (13/31)	29.79 (14/47)	34.62 (27/78)	0.333
***RASSF1*** *CpG2*	12.90 (4/31)	6.38 (3/47)	8.97 (7/78)	0.427
***HIC1***	16.13 (5/31)	8.51 (4/47)	11.54 (9/78)	0.471
***CDKN2A***	22.58 (7/31)	14.89 (7/47)	17.95 (14/78)	0.548
***CASP8***	6.45 (2/31)	2.13 (1/47)	3.85 (3/78)	0.560
***CDH13***	80.65 (25/31)	74.47 (35/47)	76.92 (60/78)	0.592
***CD44***	3.23 (1/31)	8.51 (4/47)	6.41 (5/78)	0.643
***BRCA2***	12.90 (4/31)	8.51 (4/47)	10.26 (8/78)	0.706
***RARB***	48.39 (15/31)	44.68 (21/47)	46.15 (36/78)	0.818
***APC***	45.16 (14/31)	48.94 (23/47)	47.44 (37/78)	0.819
***TIMP3***	38.71 (12/31)	36.17 (17/47)	37.18 (29/78)	1.000
***CDKN1B***	9.68 (3/31)	8.51 (4/47)	8.97 (7/78)	1.000
***VHL***	6.45 (2/31)	6.38 (3/47)	6.41 (5/78)	1.000
***PTEN***	3.23 (1/31)	4.26 (2/47)	3.85 (3/78)	1.000

*Abbreviations*: *R* recurrent disease, *NR* non recurrent disease. *P*-value < 0.05.

**Figure 2:**
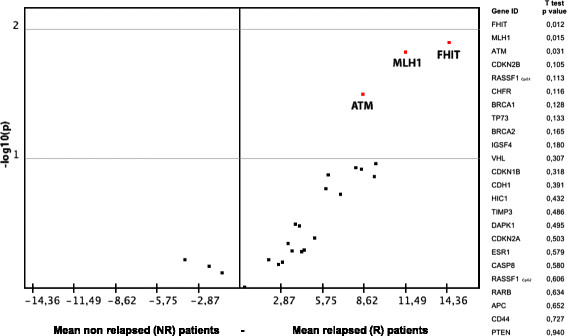
**Volcano Plot representing the differences in methylation levels between relapsed and non relapsed samples plotted against their statistical significance for all gene promoters analyzed.** The three promoters displaying significantly increased methylation levels in R samples (two-tailed *T* test, *P* < 0.05) are highlighted in the upper right corner. *T*-test *P* values of the comparison between methylation levels in R vs NR samples are shown to the right of the plot.

We then compared the mean methylation levels of gene promoters in R and NR patients, confirming that *MLH1, ATM* and *FHIT* were significantly differentially methylated in adenomas on the basis of the presence or not of lesion recurrence (Figure 2).

In particular, lower levels of methylation were associated with no recurrence of disease, while substantially higher values were correlated with relapse. Moreover, other genes showed differences in terms of methylation alterations. In particular, higher methylation levels of *CDKN2B, RASSF1, CHFR, BRCA2* and *IGSF4* were observed in adenomas that recurred.

### Methylation status phenotype and clinical pathological parameters

Taking these data into account, we evaluated the methylation status, determined on the basis of the presence or not of hypermethylation in the most significantly altered gene promoters (Table 4a,b). Analysis of *MLH1, ATM* and *FHIT* genes showed that the presence of hypermethylation in at least one of these genes indicated disease recurrence with 61% sensitivity (95% CI 44–76), and 94% specificity (95% CI 83–98), with an overall accuracy of 81% (95% CI 72–90) (Table 4a). This contrasts with the conventionally used histopathological classification which highlighted a similar distribution of recurrence in high- and low-risk subgroups (Table 2). The integration of *BRCA1* and *TP73* markers into the panel of genes did not increase accuracy when either or both were considered in methylation status analysis (Table 4b).

**Table 4:** Number of hypermethylated markers in recurrent lesions

	**Sensitivity (%)**	**Specificity (%)**	**Accuracy (%)**
	**(95%CI)**	**(95%CI)**	**(95%CI)**
**a) FHIT, MLH1, ATM**			
**≥1**	61.29 (43.82-76.27)	93.61 (82.84-97.81)	80.76 (72.02-89.52)
**≥2**	22.58 (11.40-39.81)	100 (92.44-100)	69.23 (58.99-79.47)
**≥3**	6.45 (1.79-20.72)	100 (92.44-100)	62.82 (52.09-73.55)
**b) FHIT, MLH1, ATM, TP73, BRCA1**			
**≥1**	70.96 (53.41-83.90)	85.11 (72.31-92.59)	79.49 (70.53-88.45)
**≥2**	38.71 (23.73-56.18)	95.74 (85.75-98.83)	73.08 (63.24-82.92)
**≥3**	16.13 (7.09-32.63)	100 (92.44-100)	66.66 (56.21-77.13)
**≥4**	6.45 (1.79-20.72)	100 (92.44-100)	62.82 (52.09-73.55)
**≥5**	3.22 (0.57-16.19)	100 (92.44-100)	61.53 (50.74-72.34)
**c) FHIT, MLH1**			
**≥1**	58.06 (40.77-73.58)	95.74 (85.75-98.83)	80.77 (72.02-89.52)
**≥2**	9.68 (3.35-24.90)	100 (92.44-100)	64.10 (53.45-74.75)

Sensitivity, R patients who were correctly identified by the hypermethylated profile; Specificity, NR patients who were correctly identified by the hypermethylated profile; Accuracy, R patients, correctly identified by the hypermethylated profile, and NR patients, correctly identified by the hypermethylated profile, divided by the total series; 95% CI, 95% confidence intervals.

Unconditional logistic regression analysis was carried out to evaluate the capacity of *MLH1*, *ATM* and *FHIT* gene methylation to predict recurrence. *FHIT* and *MLH1* proved to be independent variables with an RR of recurrence of 35.30 (95% CI 4.15-300.06, *P* = 0.001) and 17.68 (95% CI 1.91-163.54, *P* = 0.011), respectively. CIMP analysis showed that hypermethylation of at least 1 of these gene promoters identified recurring adenomas with 58% sensitivity and 96% specificity (Table 4c).

Methylation status was not related to age or grade of dysplasia. Conversely, a higher frequency of *MLH1* hypermethylation was associated with site of lesion. In particular, a higher frequency of methylated *MLH1* was observed in ascending with respect to descending lesions (71% and 29%, respectively, *P* = 0.07).

### Validation of MS-MLPA results

Pyrosequencing measures the methylation level of single promoter CpG sites and is used to confirm the results from other analytical methods [23].

The average methylation percentage of the same CpG sites as those used for the MS-MLPA approach was considered for data analysis (data not shown). This approach was only utilized for *MLH1* and *ATM* as reliable results were not obtained for *FHIT.* For this reason, *FHIT* was evaluated by immunohistochemistry. A methylated cut-off of ≥20% was used to discriminate between the methylated and unmethylated phenotype.

**Figure 3:**
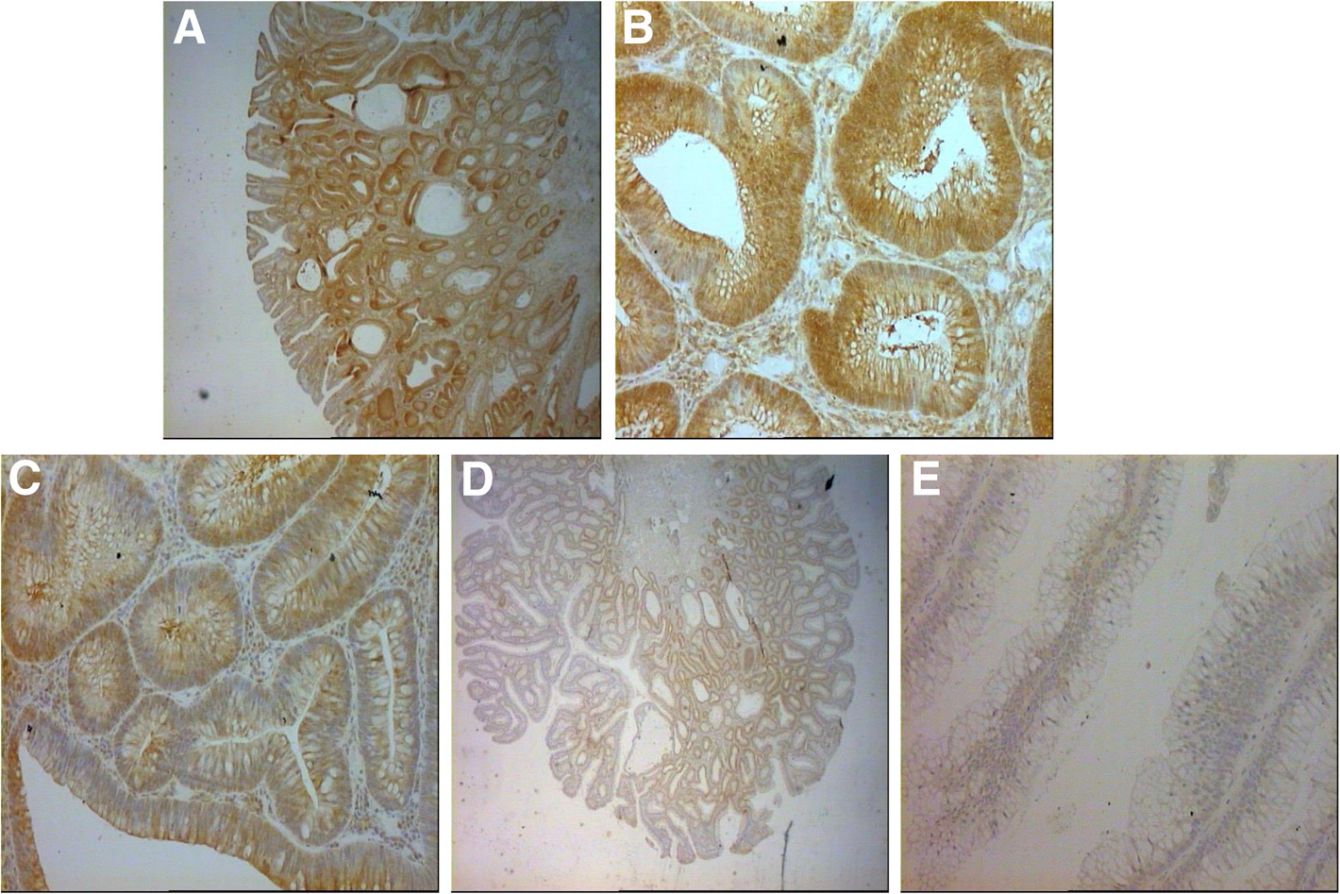
**IHC staining of FHIT protein in adenoma samples. A)** High cytoplasmic staining in 85% of colonic glands (grade 3+), a small fraction of glands (15%-20%) showing low intensity staining (grade 2+). Magnification 2.5 ×. **B)** High cytoplasmic staining in 85% of colonic glands (grade 3+). Magnification 20×. **C)** Medium cytoplasm staining in 80% of colonic glands (grade 2+). Magnification 20×. **D)** Low cytoplasmic staining in 60% of colonic glands; 40%, grade 1+ and 20%, grade 2+. Magnification 2.5×. **E)** Negative cytoplasmic staining of colonic glands. Magnification 2.5 x.

Good concordance was observed between MS-MLPA and the other two methods used (Table 1). In particular, a comparison between the MS-MLPA and pyrosequencing methods showed a 79% (57/72 cases) agreement in samples for *MLH1* and a 73% (56/77cases) agreement for *ATM,* respectively. The concordance between MS-MLPA results and IHC was 84% for *FHIT* (48/57 cases) (Figure 3). This validation was not performed on samples for which there was insufficient biological material.

## Conclusions

The adenoma-carcinoma sequence is accepted as the main pathway for the development of colorectal cancer. Although some genetic studies have provided evidence that CRC can develop in other ways, early stage CRCs frequently show adenomatous mucosa at the tumor periphery. Foci of different grades of dysplasia, intra-mucosal carcinoma and invasive cancer have also been observed in pre-neoplastic lesions, indicating a potential relationship between these different stages of colorectal lesions [7],[17]. A high number of adenomas are now detected in apparently healthy individuals undergoing routine colorectal cancer screening, but little information is available on the effective risk of recurrence in these patients.

For this purpose we selected a series of pre-neoplastic lesions classified histologically as high or low risk lesions from patients with a different clinical history. No statistically significant differences were found between adenomas classified as low risk and those classed as high risk with respect to recurrence during the 5-year follow up. Such data indicate that histopathological classification alone is insufficient to plan an adequate follow up of these patients. Moreover, grade of dysplasia, polyp size and other morphological parameters do not appear to be useful for predicting clinical evolution and therefore for organizing adequate patient surveillance. Although defined molecular subtypes of CRC exist, the molecular subgroups of CRC cannot be accurately distinguished histologically or clinically at this time [24].

Conversely, the results from the methylation profile analyzed in this study indicate that a molecular approach is capable of accurately predicting recurrence. In particular, we identified three genes (*MLH1*, *ATM* and *FHIT)* differentially methylated in adenomas that recurred during the five-year follow up. The association between the methylation of these three genes and the higher aggressiveness of pre-neoplastic lesions may be attributable to their biological functions. In fact, *MLH1* and *ATM* genes play a key role in DNA detection and repair systems and their inactivation may cause genomic DNA to become more unstable and error-prone, increasing the risk of transformation.

The MLH1 protein is involved in the DNA mismatch repair system (MMR) and methylation of this gene has been observed in CRC, especially in tumors characterized by MSI, a molecular marker of the presence of defective MMR [25],[26]. The ATM protein, a serine/threonine kinase involved in DNA double-strand break repair, is also involved in DNA repair and its inactivation is a highly destabilizing event for the cell, promoting the progression of neoplastic disease [27],[28]. It is interesting to note that *MLH1* is an independent variable, despite the molecular interaction between *MLH1* and *ATM* in regulating DNA repair*.* This suggests that concurrent inactivation of both genes may also be important in cancer development.

*FHIT*, a tumor suppressor gene involved in numerous important mechanisms associated with cell cycle response to stress signals and DNA replication control, is another independent variable [29]. Wali reported that the *FHIT* gene loses its ability to produce its specific protein in the early stages of lung, head and neck, esophageal, colorectal, breast, and cervical cancer [30]. The diminution or loss of FHIT protein expression appears to be influenced by the extensive promoter methylation program manifested in CIMP-high CRC cases [31]. *TP73* and *BRCA1* genes, both related to a higher risk of recurrence, are also involved in cell cycle control and DNA repair. In particular, *TP73* is a homolog of *TP53* tumor suppressor gene, known to be involved in the regulation of cell proliferation and apoptosis [32]-[34], while *BRCA1* represents a key regulator in the repair of double-stranded DNA breaks [26],[35]. In Huang et al.’s 2010 study on 110 stage I to IV CRC patients, *TP73* and *BRCA1* were identified from a panel of 15 radiation-related genes as prognosis-related markers on the basis of their significant correlation with clinical prognostic variables [36].

In our study, methylation status analysis of a combination of the three most significant genes (*MLH1*, *ATM*, *FHIT*) confirmed that they could be used to accurately identify patients at a higher risk of recurrence. Moreover, it is worthy of note that these genes (*MLH1*, *ATM*, *FHIT, TP73* and *BRCA1)* were not among those most frequently methylated in our case series, suggesting that the risk of recurrence is related to specific molecular characteristics.

In fact, higher aberrant methylation (more than 70% of cases with methylation levels higher than 20%) was noted for *ESR1* and *CDH13,* which are not associated with a risk of recurrence. *ESR1* has been shown to occur in histologically normal colon epithelium in an age dependent fashion [16],[37], *CDH13* is also frequently methylated in CRC and seems to be correlated with the adenoma-carcinoma transition, like other genes with methylation frequencies > 30% (*CDKN2B*, *RASSF1*, *RARB*, *APC* and *TIMP3*). Although these genes are probably related to the first step of colorectal transformation, they do not determine a molecular condition of “general colorectal instability” capable of increasing the risk of normal epithelial cell transformation. The high frequency of promoter hypermethylation of these genes confirms previously published literature data [37],[38].

The strength of our study lies in the fact that the MS-MLPA technique has the advantage of requiring a small quantity of DNA and has been shown to work well in FFPE samples [39]. However, it is also somewhat limited due to the small case series (5-year follow up records are not easily obtained in this patient setting) and to the heterogeneity of the cell population. Laser micro-dissection rather them manual macro-dissection would provide more material that is pure enough for analysis. Furthermore, when using an MS-MLPA validation approach, it must be remembered that, unlike pyrosequencing, MS-MLPA does not require bisulphate conversion and that it does not quantify the presence of protein, as does IHC.

In conclusion, a more extensive analysis is needed to confirm these preliminary data, our results would nonetheless seem to indicate that a classification based on molecular parameters could more accurately select patients at high risk of recurrence. These methylation profiles could also provide important information on the aggressiveness of the lesion and on disease evolution, useful elements when planning tailored follow up.

## Abbreviations

CRC: Colorectal cancer

FAP: Familial adenomatous polyposis

CIN: Chromosomal instability

MSI: Microsatellite instability

CIMP: CpG island methylator phenotype

MS-MLPA: Methylation-specific multiplex ligation probe analysis

dNTPs: Deoxynucleoside 5-triphosphates

FFPE: Formalin fixed paraffin-embedded

R: Relapsed

NR: Not relapsed

95% CI: 95% confidence interval

RR: Relative risks

MMR: Mismatch repair

IHC: Immunohistochemistry

## Competing interests

The authors declare that they have no competing interests.

## Authors’ contributions

CR and DC conceived and designed the study. MZ, GDM, MMT and GF carried out the immunohistochemistry assay and performed the pyrosequencing and MS-MLPA analyses. ACG and LS were responsible for patient recruitment. LS and MP interpreted the immunohistochemistry results. ES, CZ and CM performed the statistical analyses. CR, DC, GDM, MZ, GF and ES drafted the manuscript. DA and WZ reviewed the manuscript for important intellectual content. All authors read and approved the final manuscript.
